# Supplementary material for: Multi‐dataset identification of innovative feature genes and molecular mechanisms in keratoconus
Source: J Cell Mol Med. 2024 Sep 19;28(18):e70079. doi: 10.1111/jcmm.70079 (PMC11412914; doi:10.1111/jcmm.70079)
Supplement: Supplementary file 2 — Material S2. [file JCMM-28-e70079-s002.zip › 7.GSEA analysis/6.SNORA74B/KEGG_NEUROTROPHIN_SIGNALING_PATHWAY.html]

Details for gene set KEGG\_NEUROTROPHIN\_SIGNALING\_PATHWAY[GSEA]

|  || Dataset | SNORA74B.group.cls#h\_versus\_l |
| Phenotype | group.cls#h\_versus\_l |
| Upregulated in class | l |
| GeneSet | KEGG\_NEUROTROPHIN\_SIGNALING\_PATHWAY |
| Enrichment Score (ES) | -0.29182515 |
| Normalized Enrichment Score (NES) | -1.426542 |
| Nominal p-value | 0.09146342 |
| FDR q-value | 0.3132926 |
| FWER p-Value | 0.876 |
Table: GSEA Results Summary

  

Fig 1: Enrichment plot: KEGG\_NEUROTROPHIN\_SIGNALING\_PATHWAY      
 Profile of the Running ES Score & Positions of GeneSet Members on the Rank Ordered List

  

| SYMBOL | TITLE | RANK IN GENE LIST | RANK METRIC SCORE | RUNNING ES | CORE ENRICHMENT || 1 | CAMK2B | na | 141 | 0.835 | 0.0470 | No |
| 2 | PIK3R5 | na | 170 | 0.800 | 0.0951 | No |
| 3 | NGFR | na | 2599 | 0.326 | 0.0449 | No |
| 4 | CAMK2A | na | 3838 | 0.265 | 0.0253 | No |
| 5 | SOS1 | na | 5971 | 0.193 | -0.0245 | No |
| 6 | IRS4 | na | 6111 | 0.189 | -0.0170 | No |
| 7 | RPS6KA2 | na | 6719 | 0.170 | -0.0241 | No |
| 8 | CALML6 | na | 7151 | 0.158 | -0.0269 | No |
| 9 | BRAF | na | 8089 | 0.134 | -0.0458 | No |
| 10 | PIK3R1 | na | 8116 | 0.133 | -0.0384 | No |
| 11 | PLCG2 | na | 8239 | 0.131 | -0.0340 | No |
| 12 | MAPK11 | na | 8296 | 0.129 | -0.0277 | No |
| 13 | CALML5 | na | 8469 | 0.125 | -0.0250 | No |
| 14 | IKBKB | na | 8763 | 0.119 | -0.0262 | No |
| 15 | IRAK3 | na | 9124 | 0.111 | -0.0298 | No |
| 16 | TP73 | na | 9186 | 0.109 | -0.0249 | No |
| 17 | AKT2 | na | 9380 | 0.105 | -0.0240 | No |
| 18 | PLCG1 | na | 9672 | 0.099 | -0.0264 | No |
| 19 | FASLG | na | 9725 | 0.098 | -0.0219 | No |
| 20 | KRAS | na | 9766 | 0.097 | -0.0171 | No |
| 21 | IRAK1 | na | 10057 | 0.091 | -0.0199 | No |
| 22 | RPS6KA5 | na | 10419 | 0.085 | -0.0251 | No |
| 23 | MAPK9 | na | 10427 | 0.085 | -0.0201 | No |
| 24 | NTRK1 | na | 10524 | 0.083 | -0.0178 | No |
| 25 | MAPK13 | na | 11114 | 0.073 | -0.0304 | No |
| 26 | YWHAE | na | 11170 | 0.072 | -0.0276 | No |
| 27 | PSEN1 | na | 11251 | 0.071 | -0.0256 | No |
| 28 | PDPK1 | na | 11353 | 0.069 | -0.0243 | No |
| 29 | MAPK12 | na | 11855 | 0.062 | -0.0349 | No |
| 30 | CAMK2G | na | 11900 | 0.062 | -0.0325 | No |
| 31 | RPS6KA4 | na | 12098 | 0.059 | -0.0346 | No |
| 32 | CALM2 | na | 12514 | 0.053 | -0.0433 | No |
| 33 | MAPK14 | na | 12827 | 0.049 | -0.0493 | No |
| 34 | PIK3R2 | na | 12946 | 0.047 | -0.0498 | No |
| 35 | SORT1 | na | 12960 | 0.047 | -0.0473 | No |
| 36 | PIK3CA | na | 13079 | 0.046 | -0.0479 | No |
| 37 | KIDINS220 | na | 13197 | 0.045 | -0.0486 | No |
| 38 | ZNF274 | na | 13439 | 0.042 | -0.0529 | No |
| 39 | SOS2 | na | 13685 | 0.039 | -0.0576 | No |
| 40 | PRDM4 | na | 13687 | 0.039 | -0.0552 | No |
| 41 | PTPN11 | na | 13916 | 0.037 | -0.0596 | No |
| 42 | CSK | na | 14025 | 0.035 | -0.0605 | No |
| 43 | RPS6KA3 | na | 14434 | 0.031 | -0.0704 | No |
| 44 | YWHAZ | na | 14437 | 0.031 | -0.0686 | No |
| 45 | MAP3K3 | na | 14575 | 0.029 | -0.0708 | No |
| 46 | MAP2K2 | na | 14904 | 0.025 | -0.0787 | No |
| 47 | MAPK10 | na | 14996 | 0.024 | -0.0798 | No |
| 48 | CALML3 | na | 15332 | 0.021 | -0.0882 | No |
| 49 | YWHAB | na | 15465 | 0.020 | -0.0908 | No |
| 50 | PIK3CB | na | 15769 | 0.017 | -0.0985 | No |
| 51 | FRS2 | na | 16148 | 0.013 | -0.1086 | No |
| 52 | MAPK7 | na | 16513 | 0.009 | -0.1186 | No |
| 53 | NTRK3 | na | 16742 | 0.007 | -0.1247 | No |
| 54 | MAPK3 | na | 16753 | 0.007 | -0.1246 | No |
| 55 | MAP2K7 | na | 17007 | 0.005 | -0.1316 | No |
| 56 | PRKCD | na | 17017 | 0.005 | -0.1316 | No |
| 57 | RAP1A | na | 17815 | -0.004 | -0.1544 | No |
| 58 | GSK3B | na | 17871 | -0.004 | -0.1557 | No |
| 59 | RHOA | na | 18290 | -0.009 | -0.1672 | No |
| 60 | AKT1 | na | 18366 | -0.010 | -0.1688 | No |
| 61 | RAP1B | na | 18593 | -0.013 | -0.1745 | No |
| 62 | ATF4 | na | 18691 | -0.014 | -0.1765 | No |
| 63 | CDC42 | na | 18770 | -0.015 | -0.1778 | No |
| 64 | PIK3CG | na | 18884 | -0.016 | -0.1801 | No |
| 65 | MAP3K1 | na | 18950 | -0.016 | -0.1810 | No |
| 66 | BAX | na | 18953 | -0.016 | -0.1801 | No |
| 67 | BAD | na | 19265 | -0.021 | -0.1878 | No |
| 68 | TP53 | na | 19572 | -0.024 | -0.1952 | No |
| 69 | HRAS | na | 19699 | -0.025 | -0.1973 | No |
| 70 | RPS6KA6 | na | 19762 | -0.026 | -0.1975 | No |
| 71 | MAP2K5 | na | 19766 | -0.026 | -0.1960 | No |
| 72 | RAC1 | na | 19881 | -0.027 | -0.1976 | No |
| 73 | SHC3 | na | 20230 | -0.031 | -0.2058 | No |
| 74 | PIK3CD | na | 20354 | -0.032 | -0.2074 | No |
| 75 | YWHAG | na | 20368 | -0.032 | -0.2058 | No |
| 76 | MAPK8 | na | 20414 | -0.033 | -0.2051 | No |
| 77 | MAPK1 | na | 20606 | -0.035 | -0.2084 | No |
| 78 | GAB1 | na | 20982 | -0.040 | -0.2168 | No |
| 79 | RAF1 | na | 21074 | -0.041 | -0.2170 | No |
| 80 | SH2B2 | na | 21269 | -0.043 | -0.2199 | No |
| 81 | YWHAQ | na | 21272 | -0.043 | -0.2174 | No |
| 82 | ARHGDIB | na | 21720 | -0.048 | -0.2273 | No |
| 83 | NFKBIB | na | 22042 | -0.052 | -0.2334 | No |
| 84 | CALM1 | na | 22340 | -0.056 | -0.2386 | No |
| 85 | IRAK4 | na | 22391 | -0.057 | -0.2365 | No |
| 86 | TRAF6 | na | 22560 | -0.059 | -0.2378 | No |
| 87 | SHC1 | na | 22752 | -0.062 | -0.2395 | No |
| 88 | FOXO3 | na | 22782 | -0.062 | -0.2366 | No |
| 89 | CALM3 | na | 23658 | -0.075 | -0.2573 | No |
| 90 | ABL1 | na | 23665 | -0.076 | -0.2528 | No |
| 91 | ARHGDIA | na | 23902 | -0.079 | -0.2548 | No |
| 92 | IRAK2 | na | 24710 | -0.093 | -0.2724 | No |
| 93 | GRB2 | na | 24997 | -0.098 | -0.2747 | No |
| 94 | MAPKAPK2 | na | 25117 | -0.100 | -0.2720 | No |
| 95 | JUN | na | 25317 | -0.104 | -0.2714 | No |
| 96 | NGF | na | 25719 | -0.111 | -0.2761 | No |
| 97 | MAGED1 | na | 26176 | -0.121 | -0.2819 | No |
| 98 | NTF4 | na | 26519 | -0.128 | -0.2840 | Yes |
| 99 | NFKB1 | na | 26534 | -0.128 | -0.2765 | Yes |
| 100 | SH2B1 | na | 26549 | -0.129 | -0.2691 | Yes |
| 101 | CRK | na | 26740 | -0.134 | -0.2664 | Yes |
| 102 | MAP3K5 | na | 27483 | -0.151 | -0.2786 | Yes |
| 103 | RPS6KA1 | na | 27808 | -0.160 | -0.2782 | Yes |
| 104 | NTF3 | na | 27963 | -0.164 | -0.2726 | Yes |
| 105 | NRAS | na | 27969 | -0.164 | -0.2628 | Yes |
| 106 | RIPK2 | na | 28519 | -0.180 | -0.2676 | Yes |
| 107 | NFKBIA | na | 28570 | -0.182 | -0.2580 | Yes |
| 108 | RAPGEF1 | na | 28816 | -0.189 | -0.2535 | Yes |
| 109 | BDNF | na | 28985 | -0.194 | -0.2464 | Yes |
| 110 | CRKL | na | 29271 | -0.203 | -0.2423 | Yes |
| 111 | PIK3R3 | na | 29453 | -0.208 | -0.2348 | Yes |
| 112 | RELA | na | 29651 | -0.214 | -0.2274 | Yes |
| 113 | NFKBIE | na | 30232 | -0.234 | -0.2298 | Yes |
| 114 | CAMK4 | na | 30632 | -0.250 | -0.2261 | Yes |
| 115 | YWHAH | na | 30773 | -0.257 | -0.2144 | Yes |
| 116 | MAP2K1 | na | 31129 | -0.275 | -0.2079 | Yes |
| 117 | IRS2 | na | 31995 | -0.322 | -0.2131 | Yes |
| 118 | SHC4 | na | 32485 | -0.353 | -0.2057 | Yes |
| 119 | CAMK2D | na | 32655 | -0.364 | -0.1883 | Yes |
| 120 | NTRK2 | na | 33295 | -0.423 | -0.1809 | Yes |
| 121 | BCL2 | na | 33746 | -0.485 | -0.1642 | Yes |
| 122 | SH2B3 | na | 33986 | -0.529 | -0.1387 | Yes |
| 123 | SHC2 | na | 34407 | -0.652 | -0.1110 | Yes |
| 124 | AKT3 | na | 34657 | -0.880 | -0.0644 | Yes |
| 125 | IRS1 | na | 34713 | -1.087 | 0.0004 | Yes |
Table: GSEA details [plain text format]

  

Fig 2: KEGG\_NEUROTROPHIN\_SIGNALING\_PATHWAY      
 Blue-Pink O' Gram in the Space of the Analyzed GeneSet

  

Fig 3: KEGG\_NEUROTROPHIN\_SIGNALING\_PATHWAY: Random ES distribution      
 Gene set null distribution of ES for **KEGG\_NEUROTROPHIN\_SIGNALING\_PATHWAY**
